# Autologous Matrix-Induced Chondrogenesis (AMIC) for Osteochondral Defects of the Talus: A Systematic Review

**DOI:** 10.3390/life12111738

**Published:** 2022-10-29

**Authors:** Filippo Migliorini, Nicola Maffulli, Andreas Bell, Frank Hildebrand, Christian David Weber, Philipp Lichte

**Affiliations:** 1 Department of Orthopaedic, Trauma, and Reconstructive Surgery, RWTH University Hospital, Pauwelsstraße 30, 52074 Aachen, Germany; 2 Department of Orthopaedic and Trauma Surgery, Eifelklinik St. Brigida, 52152 Simmerath, Germany; 3 Department of Medicine, Surgery and Dentistry, University of Salerno, 84081 Baronissi, Italy; 4 School of Pharmacy and Bioengineering, Keele University Faculty of Medicine, Stoke on Trent ST4 7QB, UK; 5 Queen Mary Centre for Sports and Exercise Medicine, Barts and the London School of Medicine and Dentistry, Mile End Hospital, University of London, London E1 4DG, UK

**Keywords:** talus, ankle, chondral defect, cell therapies, PRP, stem cells

## Abstract

Autologous matrix-induced chondrogenesis (AMIC) has been advocated for the management of talar osteochondral lesions (OCLs). This systematic review, which was conducted according to the PRISMA 2020 guidelines, investigated the clinical and imaging efficacy and safety of the AMIC technique in the management of OCLs of the talus. Only studies investigating AMIC for talar chondral defects that were published in peer-reviewed journals were considered. In September 2022, the following databases were accessed: PubMed, Web of Science, Google Scholar, and Embase. Data on the visual analogue scale (VAS), American Orthopaedic Foot and Ankle Score (AOFAS), Tegner activity scale, and Foot Function Index (FFI) were retrieved. To evaluate the morphological MRI findings, data obtained from the magnetic resonance observation of cartilage repair tissue (MOCART) scores were evaluated. Data on hypertrophy, failures, and revision surgeries were also collected. Data from 778 patients (39% women, 61% men) were collected. The mean length of the follow-up was 37.4 ± 16.1 months. The mean age of the patients was 36.4 ± 5.1 years, and the mean BMI was 26.1 ± 1.6 kg/m^2^. The mean defect size was 2.1 ± 1.9 cm^2^. Following the AMIC technique, patients demonstrated an improved VAS (*p* < 0.001), AOFAS (*p* < 0.001), and FFI (*p* = 0.02) score. The MOCART score also improved from the baseline (*p* = 0.03). No difference was observed in the Tegner score (*p* = 0.08). No graft delamination and hypertrophy were reported in 353 patients. 7.8% (44 of 564) of patients required revision surgeries, and 6.2% (32 of 515) of patients were considered failures. The AMIC technique could be effective in improving symptoms and the function of chondral defects of the talus.

## 1. Introduction

Given its small articular surface and exposure to high loads, osteochondral lesions (OCLs) of the talus are common [1]. OCLs arise following acute ankle sprains, ligament injuries, or fractures; however, some patients present OCLs without a clear history of traumatic events [2]. OCLs can be unstable or be combined with subchondral bone defects, which may cause persistent ankle pain, impaired function, and a reduced quality of life [3,4]. Given its alymphatic, avascular, and hypocellular structure, hyaline cartilage has limited the intrinsic repair capability [5,6]. The healing process commonly results in a persistent defect or in a fibrotic scar [7,8]. In this context, a return to previous sports activity is often not achieved by conservative measures [9,10], which should typically only be considered in early stage defects (type I or II lesions according to the Berndt and Harty classification system [11]), or, given their greater regenerative potential, in children and adolescents [12]. Of the several procedures that have been developed to manage for OCLs of the talus [13], autologous matrix-induced chondrogenesis (AMIC) is a promising method [14]. The AMIC technique combines a bone stimulating procedure (drilling/microfractures) and a resorbable biologic membrane to stabilise the blood coat into the knee joint [15]. In the past decades, several investigations have reported the clinical and imaging outcomes of the AMIC technique for OCLs of the talus [16,17,18,19,20,21,22,23,24,25,26,27,28,29,30,31,32,33,34,35,36]. However, to the best of our knowledge, a comprehensive systematic review is missing. This systematic review investigated the clinical and imaging efficacy and safety of the AMIC technique in the management of OCLs of the talus.

## 2. Materials and Methods

### 2.1. Eligibility Criteria

All clinical studies investigating the AMIC technique for talar chondral defects in published peer-reviewed journals were considered. According to the authors’ language capabilities, articles in English, German, Italian, French, and Spanish were eligible. According to Oxford Centre of Evidence-Based Medicine [37], evidence from level I to IV was considered. Reviews, opinions, letters, editorials, and comments were not considered. Studies which investigated other locations of the defects rather than the talus (e.g., shoulder, hip, knee) were not considered. Studies which reported the outcomes of the AMIC technique performed in multiple locations were also not included. Animals, in vitro, biomechanics, computational, and cadaveric studies were not eligible. Only the studies which reported quantitative data under the outcomes of interest were included in the present investigation. 

### 2.2. Search Strategy

This systematic review was conducted according to the Preferred Reporting Items for Systematic Reviews and Meta-Analyses: the 2020 PRISMA statement [38]. The PICOT algorithm was preliminary elaborated:

- P (Problem): chondral defect of the talus;
- I (Intervention): AMIC;
- C (Comparison): clinical outcomes;
- O (Outcomes): PROMs and complications;
- T (Timing): minimum 12 months follow-up.

In September 2022, the following databases were accessed: PubMed, Web of Science, Google Scholar, and Embase. No time constrains were used for the search. The following keywords were used in combination using the Boolean operators AND/OR: *talus, talar, ankle, tibiotalar, chondral defects, chondropathy, cartilage defects, pain, symptoms, outcome, Autologous Matrix-Induced Chondrogenesis, AMIC, surgery, membrane, patient reported outcome measures, PROMs, complications, revision, failure*. 

### 2.3. Selection and Data Collection 

Two authors (F.M. and A.B.) independently performed the database search. All of the resulting titles were screened and, if suitable, the abstract was accessed. The full-text of the abstracts that matched the topic were accessed. If the full-text was not accessible or was not available, the article was not considered for inclusion. A cross-reference of the bibliography of the full-text articles were also performed by hand. Disagreements were debated, and the final decision was made by a third senior author (N.M.). 

### 2.4. Data Items

Two authors (F.M. and A.B.) independently performed the data extraction. The following data at baseline were extracted: author, year of publication and journal, length of the follow-up, number of patients with related mean age, and BMI. Data concerning the following PROMs were collected at baseline and at last follow-up: visual analogue scale (VAS), American Orthopaedic Foot and Ankle (AOFAS) [39], Tegner activity scale [40], and Foot Function Index (FFI) scores [41]. To evaluate the morphological MRI findings, data from magnetic resonance observation of cartilage repair tissue (MOCART) scores [42] were evaluated. Data from the following complications were also collected: hypertrophy, failures, and revision surgeries. 

### 2.5. Methodological Quality Assessment

The Coleman methodology score (CMS) was applied to assess the quality of the methodology [43]. The CMS is divided into “part A” (study size, follow-up, surgical approach, type of analysis, description of diagnosis, surgical technique, and postoperative rehabilitation) and “part B” (examining the outcome criteria and related assessment procedures and the description of subject selection process). The CMS scored the quality of the study from 0 (poor) to 100 (excellent). Final values > 60/100 are considered satisfactory. 

### 2.6. Statistical Methods

All statistical analyses were performed by the first author (F.M.). For the descriptive statistics, the means and standard deviations were used. To evaluate the improvement from baseline to last follow-up, the SPSS software package was used. The mean difference (MD) method was adopted with a 95% confidence interval (CI) and standard error (SE). The *t*-test analysis was performed with values of *p* < 0.05 being considered statistically significant.

## 3. Results

### 3.1. Study Selection

**Figure 1:**
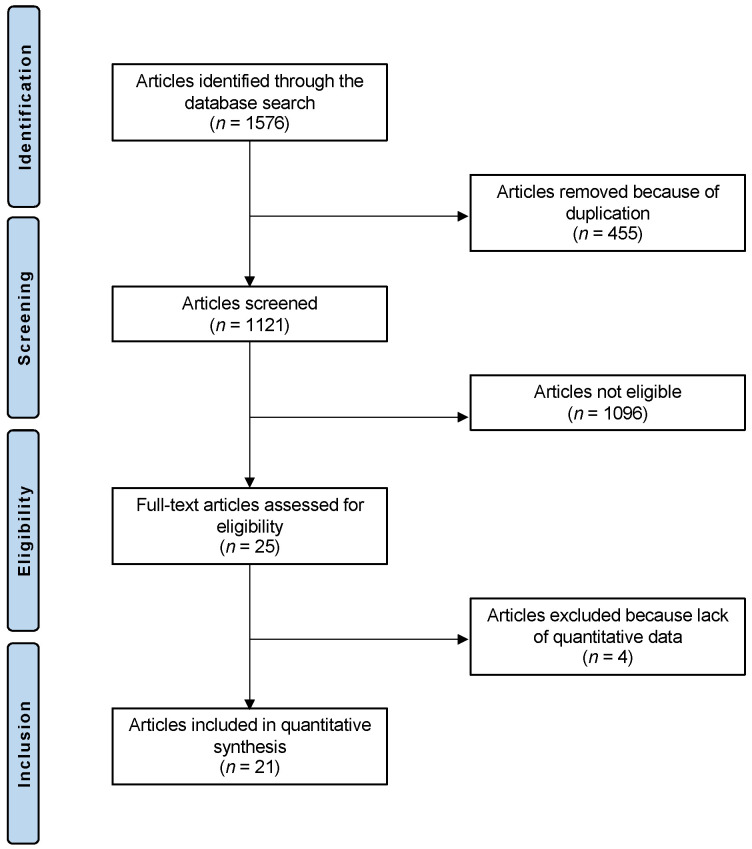
Flow chart of the literature search.

The literature search resulted in 1576 articles; of this number, 455 were duplicates, which were thus excluded. A further 1096 articles were excluded for the following reasons: study type (*n* = 401), other location rather than talus (*n* = 279), mixed location (*n* = 9), not focused on chondral defects (*n* = 405), and language limitation (*n* = 2). A further four articles were excluded as they did not report quantitative data under the outcomes of interest. This left 21 articles for inclusion: five prospective and sixteen retrospective clinical studies. The literature search results are shown in Figure 1.

### 3.2. Methodological Quality Assessment

The retrospective and unblinded nature of most of the included studies represent an important limitation. The study size and length of the follow-up were determined to be appropriate in most studies. The descriptions of the diagnoses, surgical procedures, and postoperative rehabilitation protocols were adequately reported by most studies. The outcome measures and timing of assessment were often defined, providing moderate reliability. General health measures were seldom reported. The procedures for assessing the outcomes and subject selection were often biased and not satisfactorily described. In conclusion, the CMS score resulted in a 62/100, attesting a good quality of methodological assessment to this review study. The CMS score of each included study is reported in Table 1.

### 3.3. Study Characteristics and Results of Individual Studies

Data from 778 patients (39% women, 61% men) were collected. The mean length of the follow-up was 37.4 ± 16.1 months. The mean age of the patients was 36.4 ± 5.1 years, and the mean BMI was 26.1 ± 1.6 kg/m^2^. The mean defect size was 2.1 ± 1.9 cm^2^. The generalities and demographic of the included studies is shown in Table 1.

### 3.4. Efficacy of AMIC

Patients who underwent the AMIC technique demonstrated an improved VAS (*p* < 0.001), AOFAS (*p* < 0.001), and FFI (*p* = 0.02) score. The MOCART score was also improved from the baseline (*p* = 0.03). No difference was evidenced in the Tegner score (*p* = 0.08). These results are shown in greater detail in Table 2.

### 3.5. Complications

No signs of graft delamination and hypertrophy were reported in 353 patients. It was observed that 7.8% (44 of 564) of patients experienced a revision surgery, and 6.2% (32 of 515 of patients) were considered failures. 

## 4. Discussion

According to the main findings of the present study, the AMIC technique could be effective in improving the symptoms and function in patients with OCLs of the talus. Statistically significantly decreases in pain and enhancements of ankle function (AOFAS/FFI) were observed. At imaging, the MOCART score also confirmed an improvement from the baseline. No graft delamination and hypertrophy were reported in any of the included studies. The rate of revision surgery and surgical failure were 7.8% and 6.2%, respectively. Though these complications are not infrequent, these data may well be biased and underestimated. Indeed, most authors did not clearly report whether complications, including failures, had occurred. 

Several surgical modalities have recently been used to manage OCLs of the talus, including the fixation of osteochondral fragments to restore hyaline articular cartilage and natural congruency, bone marrow stimulation (BMS) techniques (microfractures, nanofractures, microdrilling) to stimulate fibrocartilaginous repair, autologous osteochondral transplantation (OAT), and autologous chondrocyte implantation (ACI). In patients who present subchondral bone damage, autologous bone transplantation from the malleolar osteotomy or from the iliac crest is commonly performed [44]. Unfortunately, there is a lack of head-to-head comparative studies and, above all, RCTs, regarding these techniques.

Given their simple execution and good results, the BMS technique has been commonly performed for the management of talar OCLs, though the blood clot after isolated BMSs might not stable enough in larger defects. Nevertheless, these procedures have a limited potential in lesions larger than 1.5 cm^2^ [45]. The size of the lesion significantly correlates with the clinical outcome [46]. However, a gold standard for the management of the OCLs of the talus has not yet been established [4]. ACI techniques have been commonly used in OCLs of the talus [47]; however, they are performed as a two-session surgery and are burdened from the harvesting of healthy cartilage from a non-weight bearing zone of the knee [48]. Moreover, ACI requires laboratory expansion of the harvested chondrocytes [48]. In this context, the AMIC technique has gained increasing interest. The AMIC approach is a BMS procedure performed in single surgical session [32]. After debridement of the non-viable chondral tissue, the subchondral bone is microfractured, and the membrane is placed into the defect [32]. This collagen matrix helps to retain the blood clot, including the bone marrow-derived mesenchymal stem cells (BM-MSCs), in the defect zone (“biological chamber”) [49]. The membrane also protects the blood coating from the articular environment and shear forces [50]. Collagen is additionally believed to enhance the BM-MSCs proliferation and differentiation [51]. These features cause the AMIC technique to be of special interest [52]. There is a consensus in the International Consensus Group on Cartilage Repair that, for the surgical management of OCLs > 1 cm², the augmentation of a scaffold leads to better and more reliable results [53]. Other authors have suggested that OCLs > 1.5 cm² required a membrane scaffold [54]. In the present investigation, the mean defect size was 2.1 ± 1.9 cm^2^, which represents the optimal size for the AMIC technique. The good functional results support that the AMIC technique might be promising even in larger defects of the talus. Likewise, Kubosch et al. reported that us of the AMIC technique in large lesions with an average size of 2.4 cm² showed comparable results to smaller lesions [28]. In contrast to the improvement in pain and specific foot scores, the activity levels seem to be not significantly increased after the operation. This is in line with a study by Wiewiorski et al., who showed that even after morphologically successful operations, the sports activity level was only slightly increased in comparison to the preoperative status [35]. This underlines the recommendation that only painful OCLs of the talus should be operated on, especially because there is no evidence that OCLs of the talus increase the risk for ankle arthritis in a way that justifies prophylactic operation in asymptomatic patients [55]. MRI scans often continue to be evidence abnormalities following the AMIC technique [28], with more than 90% of patients who had underwent AMIC operations showing local subchondral oedema and irregularities of the subchondral lamella 5 years after the index procedure [24]. In the MRI scans, there was no difference in the cartilage signal between the reconstructed tissue and healthy cartilage following the AMIC technique in spite of a persistent bone oedema [28]. The increase in the MOCART score in our study suggests an improvement of the cartilage after AMIC operation at imaging. However, given the lack of association with a clinical outcome, the MOCART score must be critically evaluated [56].

The present investigation has some limitations. Given the lack of larger prospective studies, only retrospective studies with a relatively small number of patients and procedures have been included in the present investigation. The grey literature was not accessed to identify additional studies, as it should not contribute to scientific analyses and recommendations. Most authors did not clearly state the number of patients who required bone grafting and their related harvesting procedure; therefore, further analyses of subgroups were not possible. The impact of subchondral bone necrosis on the surgical outcome has not yet been fully clarified, and further investigations are required. The surgical procedure as well as the postoperative rehabilitation were often not precisely described and were not consistent between the studies. One important variation is that different techniques were used to obtain subchondral blood have been used in the AMIC operations. Microfractures as described by Steadman et al. [57] have limitations in the depth of subchondral access of the microfracture awl and damages the subchondral bone plate by trabecular compaction around the perforation. These are currently thought to be among the causes of the fibrocartilaginous tissue formation associated with this technique [58]. Therefore, nanofracturing [59] and microdrilling were developed using smaller needles and deeper subchondral access (9 mm), supposedly to minimize the destruction of the subchondral bone plate in comparison to microfractures [59,60]. Accordingly, deep drilling (6 mm) was superior to shallow drilling (2 mm) and microfractures [61,62]. Whether those different effects on the migration of BM-MSCs and subchondral bone stock exert an influence on the clinical outcome of the AMIC technique is unknown. Moreover, two different membranes were used: Chondro-Gide (Geistlich Pharma AG, Wolhusen, Switzerland) and Cartimaix (Matricel GmbH, Herzogenrath, Germany). Most evidence arises from the Chondro-Gide^®^ membrane, but there are no comparative studies between these membranes. Most authors fixed the membrane through the application of fibrin glue into the defect; however, some authors did not state how the fixation was achieved. Whether gluing influences cartilage regeneration has not yet been fully clarified. In a recent in vitro study, fibrin glue over a resorbable membrane commonly employed in AMIC operations impairs the proliferation and migration of chondrocytes [63]. With its additional advantages, such as cost effectiveness and preservation of healthy cartilage, our data support that the AMIC technique is safe in talar OCLs, despite the lack of studies concerning the long-term outcome. Furthermore, additional studies are necessary to more precisely define the critical defect size.

## 5. Conclusions

According to the main findings of the present study, the AMIC technique appears effective in improving the symptoms and function of OCLs of the talus.

## Figures and Tables

**Table 1:** Generalities and patient baseline of the included studies (CMS: Coleman Methodology Score).

Author, Year	Journal	Design	CMS	Follow-Up (Months)	Patients (*n*)	Women (*%*)	Mean Age	Mean BMI
Ackermann et al. 2021 [16]	*Orthop. J. Sports Med.*	Retrospective	61	50.4	13		33.3	26.2
				50.4	13		33.4	26.1
Albano et al. 2017 [17]	*BMC Muskulos. Dis.*	Retrospective	54	30.0	16	50	42.6	26.3
Ayyaswamy et al. 2021 [18]	*Foot Ankle Surg.*	Retrospective	66	24.0	25	44	36.0	
Baumfeld et al. 2018 [19]	*Foot*	Retrospective	50	10.8	17	47	37.5	
Becher et al. 2018 [20]	*Knee Surg. Sports Traumatol. Arthrosc.*	Retrospective	54	68.4	16	56	32.4	22.6
D’Ambrosi et al. 2017 [21]	*Arthroscopy*	Retrospective	53	27.0	17	53	25.0	
					14	26	47.0	
D’Ambrosi et al. 2019 [22]	*Clin. J. Sport Med.*	Retrospective	64	42.6	26	35	33.7	24.5
Galla et al. 2018 [23]	*Knee Surg. Sports Traumatol. Arthrosc.*	Retrospective	55	33.5	23	35	35.6	
Gottschalk et al. 2017 [24]	*J. Foot Ankle Surg.*	Retrospective	67	60.0	21	38	37.0	26.0
Goetze et al. 2021 [25]	*Life*	Prospective	77	25.2	24	50	46.8	26.9
Goetze et al. 2021 [26]	*BMC Muskulos. Dis.*	Prospective	77	66.2	19	53	47.3	24.1
Kretzschmarr et al. 2014 [27]	*Eur. Radiol.*	Prospective	64		25	32	38.0	28.0
Kubosch et al.2015 [28]	*Int. Orthop.*	Retrospective	56	39.5	17	47	38.8	27.4
Migliorini et al. 2021 [29]	*Life*	Prospective	79	44.2	52	0	31.5	27.1
				41.5	18	44	33.3	6.9
Richter et al. 2019 [30]	*Foot Ankle Surg.*	Prospective	74	24.4	129	41	35.3	
				23.8	129	40	35.6	
Usuelli et al. 2016 [31]	*Knee Surg. Sports Traumatol. Arthrosc.*	Retrospective	64	24.0	20	45	36.1	24.6
Valderrabano et al. 2013 [32]	*Am. J. Sports Med.*	Retrospective	67	30.9	26	31	34.6	
Weigelt et al. 2019 [33]	*Am. J. Sports Med.*	Retrospective	56	56.4	33	42	35.1	26.8
Wiewiorski et al. 2013 [34]	*Clin. Radiol.*	Retrospective	51	23.3	23	30	34.2	28.5
Wiewiorski et al. 2016 [35]	*Am. J. Sports Med.*	Retrospective	62	46.9	60	40	34.9	27.6
Yontar et al. 2018 [36]	*Acta Orthop. Traumatol. Turc.*	Retrospective	59	20.3	20	30	32.9	

**Table 2:** Improvements of the PROMs from the baseline to the last follow-up (FU: follow-up; MD: mean difference; CI: confidence interval; AOFAS: American Orthopaedic Foot and Ankle Score; FFI: Foot Function Index; MOCART: magnetic resonance observation of cartilage repair tissue).

Endpoint	Baseline	Last FU	MD	SE	95% CI	*p*
Visual Analogue Scale	7.1 ± 1.2	2.2 ± 0.7	−5.0	0.1	−4.9 to −4.8	<0.001
Tegner Activity Scale	3.3 ± 0.6	4.2 ± 0.7	0.9	0.0	0.8 to 0.9	0.08
AOFAS	54.2 ± 8.3	86.3 ± 5.5	32.2	0.4	31.3 to 32.8	<0.001
FFI	49.6 ± 4.3	29.6 ± 12.4	−20.0	0.5	−20.9 to −19.0	0.02
MOCART	39.3 ± 9.9	59.6 ± 11.9	20.3	0.6	19.2 to 21.3	0.03

## Data Availability

The datasets generated during and/or analysed during the current study are available throughout the manuscript.
